# Preclinical evaluation of cancer immune therapy using patient‐derived tumor antigen‐specific T cells in a novel xenograft platform

**DOI:** 10.1002/cti2.1246

**Published:** 2021-02-02

**Authors:** Gautam N Shenoy, Christopher J Greene, Maulasri Bhatta, Miren L Baroja, Jenni L Loyall, Sathy V Balu‐Iyer, Raymond J Kelleher, Beatriz M Carreno, Gerald P Linette, Leonard D Shultz, Richard B Bankert

**Affiliations:** ^1^ Department of Microbiology and Immunology at the Jacobs School of Medicine and Biomedical Sciences University at Buffalo Buffalo NY USA; ^2^ Immune Modulatory Therapies, LLC Eden NY USA; ^3^ Center for Cellular Immunotherapies Perelman School of Medicine University of Pennsylvania Philadelphia PA USA; ^4^ Department of Pharmaceutical Sciences University at Buffalo Buffalo NY USA; ^5^ The Jackson Laboratory Bar Harbor ME USA; ^6^ Present address: Hodgson Russ LLP. Buffalo NY USA; ^7^ Present address: Roswell Park Comprehensive Cancer Center Buffalo NY USA

**Keywords:** cancer immunotherapy, checkpoint blockade, melanoma, patient‐derived xenograft, T cells

## Abstract

**Objectives:**

With a rapidly growing list of candidate immune‐based cancer therapeutics, there is a critical need to generate highly reliable animal models to preclinically evaluate the efficacy of emerging immune‐based therapies, facilitating successful clinical translation. Our aim was to design and validate a novel *in vivo* model (called Xenomimetic or ‘X’ mouse) that allows monitoring of the ability of human tumor‐specific T cells to suppress tumor growth following their entry into the tumor.

**Methods:**

Tumor xenografts are established rapidly in the greater omentum of globally immunodeficient NOD‐*scid IL2Rγ^null^* (NSG) mice following an intraperitoneal injection of melanoma target cells expressing tumor neoantigen peptides, as well as green fluorescent protein and/or luciferase. Changes in tumor burden, as well as in the number and phenotype of adoptively transferred patient‐derived tumor neoantigen‐specific T cells in response to immunotherapy, are measured by imaging to detect fluorescence/luminescence and flow cytometry, respectively.

**Results:**

The tumors progress rapidly and disseminate in the mice unless patient‐derived tumor‐specific T cells are introduced. An initial T cell‐mediated tumor arrest is later followed by a tumor escape, which correlates with the upregulation of the checkpoint molecules programmed cell death‐1 (PD‐1) and lymphocyte‐activation gene 3 (LAG3) on T cells. Treatment with immune‐based therapies that target these checkpoints, such as anti‐PD‐1 antibody (nivolumab) or interleukin‐12 (IL‐12), prevented or delayed the tumor escape. Furthermore, IL‐12 treatment suppressed PD‐1 and LAG3 upregulation on T cells.

**Conclusion:**

Together, these results validate the X‐mouse model and establish its potential to preclinically evaluate the therapeutic efficacy of immune‐based therapies.

## Graphical abstract

We have designed and validated a first‐of‐its‐kind xenograft model, the Xenomimetic Mouse (X‐mouse) model, that has the ability to rapidly and reliably evaluate immune‐based therapies for cancer treatment using patient‐derived tumor‐specific T cells. Our model offers multiple advantages over existing preclinical models including but not limited to easy, accurate and quick measurement of tumor burden, monitoring changes in T‐cell phenotypes at the single cell level and rapid turnaround times. By validating the X‐mouse model using established clinically relevant immune‐based therapies, we demonstrate that it has potential to be a robust preclinical tool that will be of great utility to investigators involved in the development of immunotherapeutic targets and therapeutics for cancer treatment.

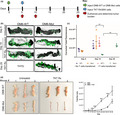

## Introduction

The emergence of cancer immunotherapy in the last decade, illustrated by advances in adoptive cellular therapy and immune cell‐targeted monoclonal antibody (mAb) therapy, has resulted in a vast improvement in the overall survival of patients with advanced‐stage cancer.^1^ This has understandably generated a lot of interest in the field, resulting in a plethora of candidate immunotherapeutic targets and therapeutics for preclinical testing, which is usually carried out in animal models. Successful translation of preclinical results is predicated upon how clinically predictive these models are. Findings that over 90% of new drugs are ineffective in clinical trials^2^, ^3^ suggest that the inefficiency of current preclinical models contributes towards the low translation rate. There is therefore an unmet critical need for the design and validation of animal models to preclinically evaluate the efficacy of immune‐based therapies.

While cell line‐derived xenograft models are more commonly used for initial phases of drug development and toxicity studies, patient‐derived xenograft (PDX) models, which involve implantation of cancer tissue from individual patients into immunodeficient mice, are more reliable models for preclinical research. Starting from the first successful engraftment of human tumors into immunodeficient mice over 30 years ago,^4^ a number of xenograft models have been reported^5^, ^6^, ^7^, ^8^ with attempts made to assess the efficacy of immunotherapies.^7^, ^9^, ^10^, ^11^, ^12^ More recently, humanised NOD‐*scid IL2Rγ^null^* (NSG) mice (HuNSG) mice were developed by implanting hematopoietic stem and progenitor cells into conditioned NSG mice, resulting in the generation of multiple human immune cells including T cells, B cells, plasma cells, dendritic cells and myeloid cells.^13^, ^14^, ^15^ These HuNSG mice, which develop a partially functional immune system,^7^, ^13^, ^16^ overcome some limitations of the earlier PDX models. While this approach has great potential for evaluating immune‐based strategies,^17^, ^18^ it is logistically challenging, requiring up to 12 weeks to generate these functional immune cells, a further 60 days to establish tumor xenografts and several additional weeks to assess the response of the T cells to tumors. In general, PDX models, including the humanised mouse model, have been logistically challenging and difficult to standardise as it has not been possible in most cases to control the number of tumor‐specific T cells in the xenografts or to confirm and to identify the tumor specificity of the T cells in the model.^17^, ^18^

Here, we report a novel mouse model, the Xenomimetic mouse (X‐mouse) model, established using patient‐derived tumor‐specific T cells and GFP^+^ melanoma tumor target cells expressing melanoma patient‐derived tumor neoantigen peptides in the context of matched HLA. The use of a defined number of tumor target cells and adoptively transferred tumor antigen‐specific patient‐derived CD8^+^ T cells makes it possible to monitor human anti‐tumor T‐cell responses in a controlled environment in the X‐mouse model. We validate the ability of our model to rapidly evaluate the therapeutic efficacy of immune‐based therapies designed to enhance the anti‐tumor potential of tumor‐specific T cells following their entry into the tumor microenvironment. We conclude that the X‐mouse model represents a reliable preclinical platform to evaluate the efficacy of new immune‐based therapies for cancer as standalone treatments or combination therapies.

## Results

### Generation of the Xenomimetic (X) mouse model

The X‐mouse model consists of two cellular components: melanoma tumor target cells and tumor‐specific T cells. These cells were generated and characterised previously as part of a neoantigen vaccination clinical trial with melanoma patients.^19^, ^20^ Tumors resected from stage III melanoma patients enrolled in the trial were exome and transcriptome sequenced to identify expressed tumor‐specific mutated proteins. *In silico* and algorithmic analysis was performed to predict high‐affinity HLA‐A*02:01 binding amino acid substituted peptides arising from identified mutated proteins. Synthetic peptides were manufactured and used to immunise melanoma patients with peptide‐pulsed autologous dendritic cells as part of a personalised neoantigen vaccination. Each melanoma patient received a unique set of peptides selected based on tumor mutational profile. After vaccination, CD8^+^ T cells specific for amino acid substituted peptides were isolated from patients’ leukapheresis products, expanded *in vitro* and sorted using custom HLA/peptide dextramers to obtain 70–95% enriched neoantigen‐specific T‐cell populations. Tumor‐specific cells used in our study are TKT R438W and TMEM48 F169L, derived from patient MEL21, and a detailed characterisation of these T cells has been previously reported^19^ (Supplementary figure 1).

To generate tumor target cells, DM6, an HLA‐A*02:01^+^ melanoma cell line, was transfected with tandem minigene constructs (TMCs) encoding the mutated neoantigen peptides from patient MEL21 (DM6‐Mut) or the corresponding wild‐type peptides (DM6‐WT). The TMCs were cloned into vectors that encode GFP, which allows easy detection of tumor cells.^19^ The expression of the respective peptide in the context of HLA‐A*02:01 as well as GFP in DM6‐WT and DM6‐Mut cells was confirmed.^19^

**Figure 1:**
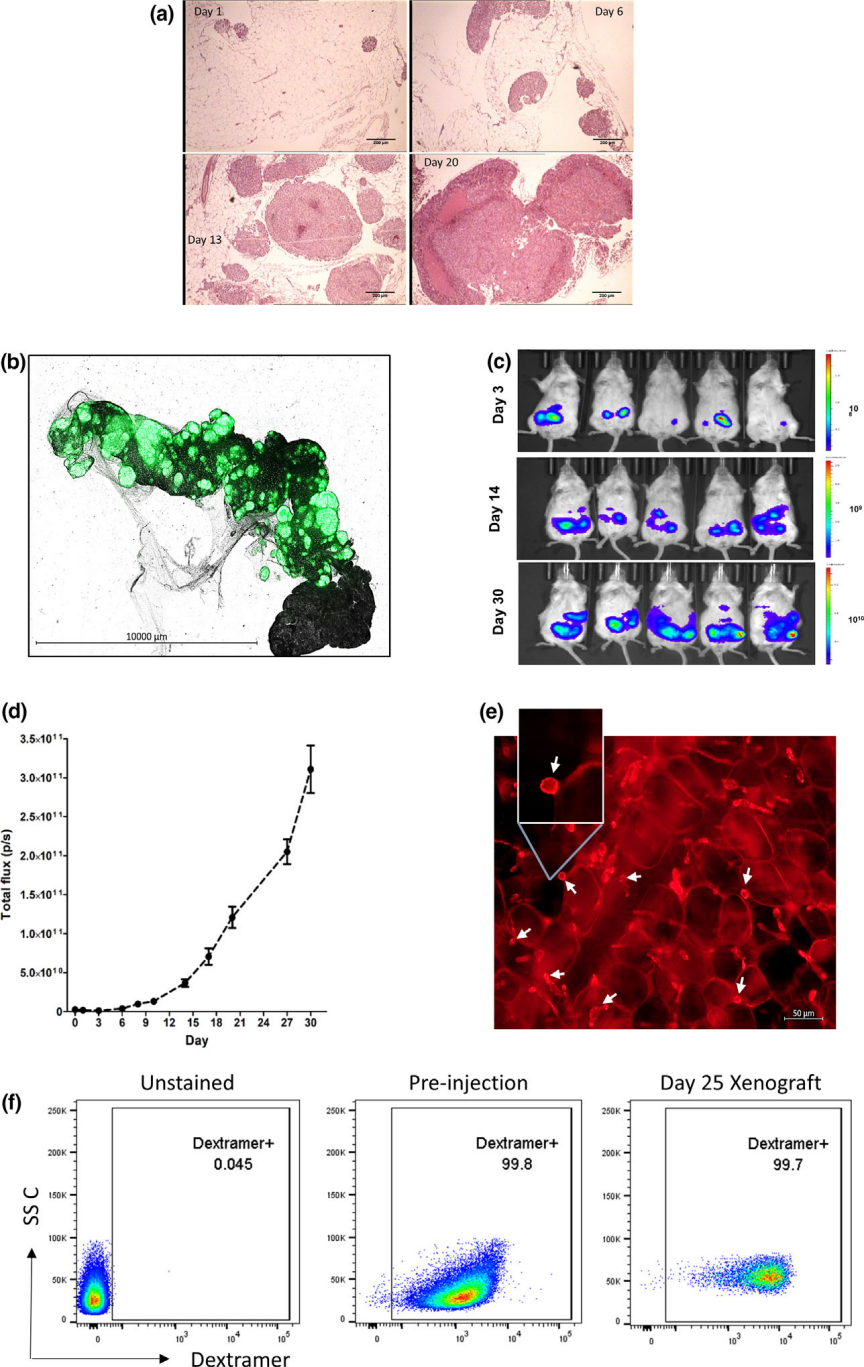
The Xenomimetic mouse model supports the growth of xenogeneic tumor cells and tumor‐specific T cells. **(a)** 2.5 × 10^6^ DM6‐Mut cells were injected i.p. into NSG mice. The mice were euthanised on days 1, 6, 13 and 20, and the greater omentum was harvested. Tumor burden was visualised by H&E staining of the omental sections. Scale bar = 200 µm. **(b)** Tumors are visualised as green (GFP^+^) areas in omental whole mounts on day 11. **(c, d)** Luciferase^+^ DM6 tumors can be visualised and quantified by live imaging. Xenografts were established using 2.5 × 10^6^ DM6‐Mut‐LUC^+^ cells. On different days following the injection of tumors, luciferin was injected and photon flux quantified to determine tumor burden. Representative images of DM6 tumors on different days are shown in **c**. Quantified tumor burdens over time (*n* = 5 mice per group) are shown in **d**. **(e)** 5 × 10^6^ TKT R438W cells were injected i.p. into NSG mice. The mice were euthanised on day 6, and the greater omentum was harvested. A whole mount of the omentum was stained with AlexaFluor 647‐conjugated anti‐human CD3 and scanned under a fluorescence microscope. CD3^+^ T cells are denoted by white arrows. **(f)** 3 × 10^6^ TKT R438W cells were injected on day 5 into DM6‐Mut xenografts and tumor‐specific T cells (TKT R438W) detected on day 25 by identifying binding to peptide‐loaded dextramers. Gated on live CD3^+^CD8^+^ cells (*n* = 4 mice per group).

The animal component of the X‐mouse model is a strain of globally immunodeficient mice, i.e. the NSG mice. Intraperitoneal (i.p.) injection of DM6 cells into NSG mice results in their rapid and preferential engraftment to an anatomically well‐defined site, the greater omentum. The greater omentum is a small strip of well‐vascularised fatty tissue located between the stomach, pancreas and spleen that has been shown to support the growth of multiple patient‐derived tumors including ovarian tumors^9^ and lymphomas.^21^ Pockets of DM6 tumor cells easily distinguishable from the omental fat cells are visible by haematoxylin and eosin (H&E) staining of omental sections as early as 1 day following injections, and these tumors grow rapidly over time (Figure 1a). More importantly, the presence of GFP as a reporter enables scanning of whole mounts of the entire omentum under a fluorescence microscope to view and accurately quantify (GFP^+^) tumor burden post‐mortem (Figure 1b).

To enable detection and quantitation of tumor growth and spread across multiple organs, and also to track tumor growth kinetics in the same animals, we transduced DM6‐WT and DM6‐Mut cells with firefly luciferase (Supplementary figure 2). Live imaging of mice‐bearing luciferase‐expressing DM6 cells following the injection of luciferin allows determination of tumor burdens in the same animals over time. As observed in the omentum, robust growth of DM6 cells was seen in these mice with a concomitant spread over time (Figure 1c and d).

After establishing the growth of tumor in the omentum, we sought to determine whether tumor‐specific TKT R438W cells injected i.p. can localise and survive in the greater omentum. We detected CD3^+^ T cells 6 days following their i.p. injection, establishing their presence in the omentum (Figure 1e). Additionally, we detected the presence of viable antigen‐specific CD8^+^ T cells (dextramer^+^CD3^+^CD8^+^ cells) in day 25 DM6‐Mut xenografts, establishing that adoptively transferred T cells survive and persist in the xenografts (Figure 1f). Together, these results confirmed that the omentum can harbour and support the growth of xenogeneic tumor cells and tumor‐specific T cells.

### Preclinical therapeutic efficacy of adoptively transferred tumor‐specific T cells demonstrated using the X‐mouse model

**Figure 2:**
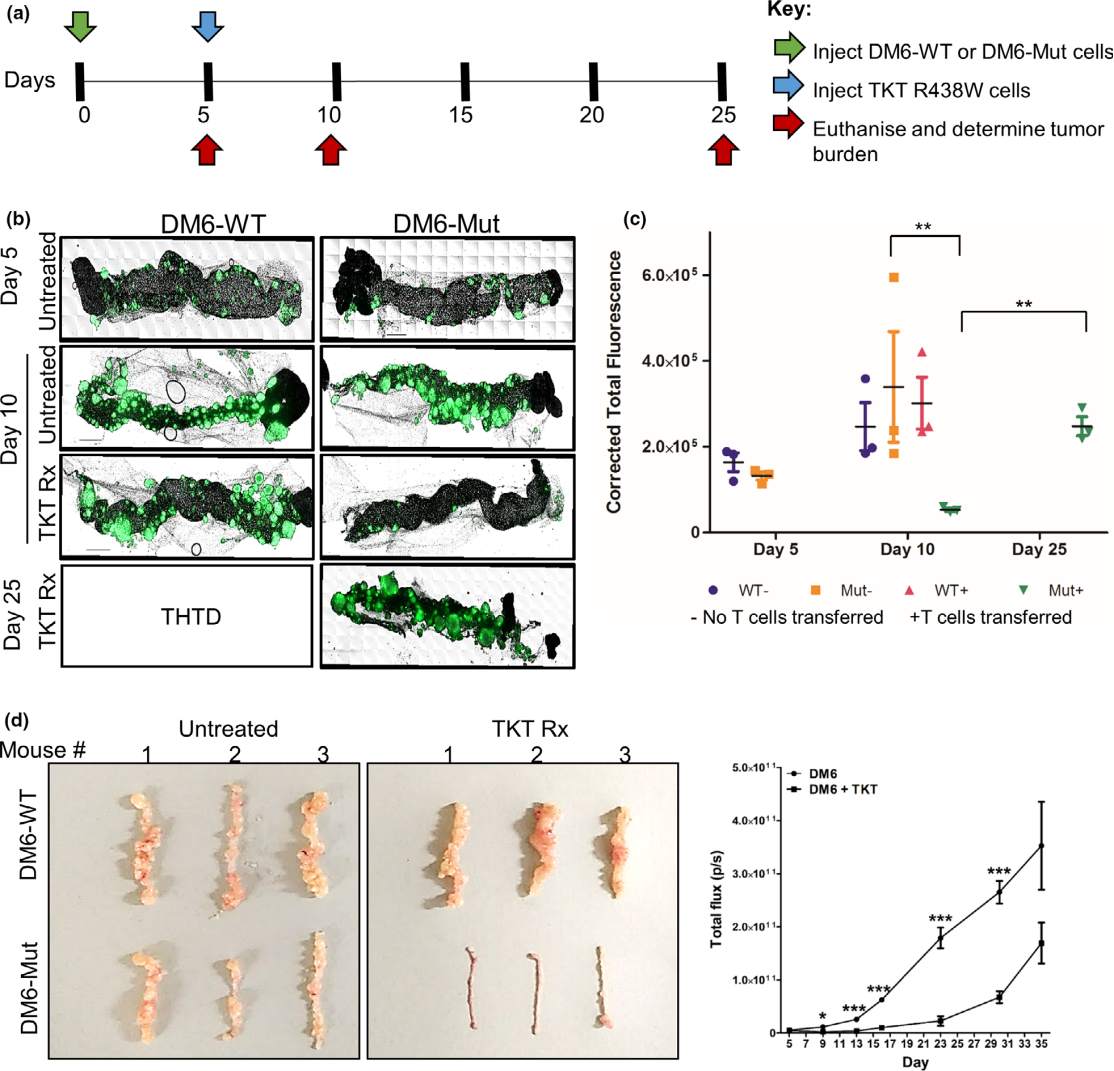
Initial suppression of tumor growth following the adoptive transfer of tumor‐specific T cells is followed by tumor escape in the X‐mouse model. **(a)** Experimental scheme indicating the timeline for injection of tumor cells (green arrow), TKT R438W cells (blue arrow) and estimation of tumor burden (red arrows). **(b)** Representative images of omental tumor burdens on days 5, 10 and 25. **(c)** Tumor burdens were determined by quantifying the GFP signal using ImageJ software and are represented as corrected total fluorescence (CTF). There are no bars for WT−, WT+ and Mut‐ cohorts on day 25 as they were THTD (*n* = 3 mice per group). **(d)** Gross images of omenta on day 25 from different groups. **(e)** Xenografts established using 2.5 × 10^6^ DM6‐Mut cells were either left untreated, or treated with 1 × 10^6^ TKT R438W cells on day 5. Tumor burden was determined on different days by measuring the photon flux following the i.p. injection of luciferin (*n* = 5 mice per group). Data are presented as mean ± SEM. *P ≤ 0.05; **P ≤ 0.01, ***P ≤ 0.001. THTD = too high to determine accurately.

TKT R438W cells have been shown *in vitro* to recognise antigen (mutated TKT R438W peptide) specifically and with high avidity. *In vitro* studies have also shown evidence of significant cytotoxicity by these cells towards target cells expressing endogenously processed and presented peptide in the context of HLA‐A*02:01.^19^ To test the therapeutic efficacy of TKT R438W cells in suppressing the growth of DM6 cells *in vivo*, 1 × 10^6^ TKT R438W cells were injected i.p. into 5‐day‐old xenografts established with either DM6‐Mut cells, which express the antigen (mutated TKT R438W peptide) in the context of HLA‐A*02:01, or control DM6‐WT cells, which express the wild‐type (nonmutated TKT) peptide (Figure 2a). Tumor burdens were determined *post‐mortem* by microscopic scanning of omental whole mounts and quantifying the GFP signal in the omenta. Comparison of tumor burdens on day 10 (i.e. 5 days following transfer of TKT R438W cells) revealed significant suppression of DM6‐Mut, but not DM6‐WT cells (Figure 2b and c), demonstrating the specificity of TKT R438W cells, as well as their cytotoxicity *in vivo*. Upregulation of the activation marker CD69 on T cells isolated from day 10 DM6‐Mut xenografts confirmed that these cells are activated upon entry into the tumor microenvironment (Supplementary figure 3).

### Tumors escape from T‐cell control in the X‐mouse model

To determine the durability of this T cell–mediated tumor suppression, we measured tumor burdens on day 25 in the above cohorts of mice. When left untreated, mice with DM6‐Mut xenografts show rapid tumor growth, and by day 25, the tumor burden in the omenta is too high to be accurately quantified using microscopic scanning (too high to determine accurately or THTD). Gross images are shown for these mice (Figure 2d). Moreover, these mice also develop peritoneal ascites fluid and metastatic lesions on the liver, spleen and in the peritoneum. Of particular importance to our model is our observation that the initial T‐cell suppression of tumor on day 10 is followed later by a tumor escape from T‐cell control. The tumor escape is consistently observed by day 25 as evidenced by a significant increase in tumor burden (Figure 2b and c). Control untreated DM6‐WT xenografts were grossly indistinguishable from TKT R438W‐treated DM6‐WT xenografts on day 25 (Figure 2d).

The escape of tumors following initial suppression observed in the omentum was also demonstrated using *in vivo* imaging to quantify tumor burdens in the entire mouse (Figure 2e). Injection of TKT R438W cells on day 5 suppresses growth of DM6‐Mut‐LUC^+^ cells, which begin to escape the T‐cell control by day 23.

Transferring escalating doses of TKT R438W cells (ranging from 1 × 10^4^ cells to 3 × 10^6^ cells) resulted in a dose‐dependent suppression of tumor burdens on day 10 (Supplementary figure 4 and Supplementary table 1). However, it could not prevent the escape of DM6‐Mut tumors from T cell control on day 25, although the degree of tumor escape inversely correlated with the numbers of adoptively transferred T cells. This suggested that tumor recurrence was not simply a matter of insufficient T‐cell numbers, and led us to predict and test the probability that the tumor escape from T‐cell control may be due in part to immunosuppressive mechanisms within the microenvironment of the tumor xenograft.

### The X‐Mouse model as a tool for preclinical testing of adoptive T‐cell therapy protocols

We postulated that the kinetics of tumor escape could also be used to determine the efficacy of altering the adoptive T‐cell therapy protocol and timing of the T‐cell transfer. This was tested by determining whether the tumor escape could be delayed or prevented by the adoptive transfer of a combination of T cells specific for different tumor antigens or by the repeated injection of T cells specific for one tumor neoantigen. The answer to these questions would be of potential value in designing the most therapeutically effective adoptive cell transfer for cancer patients using tumor antigen‐specific T cells.

**Figure 3:**
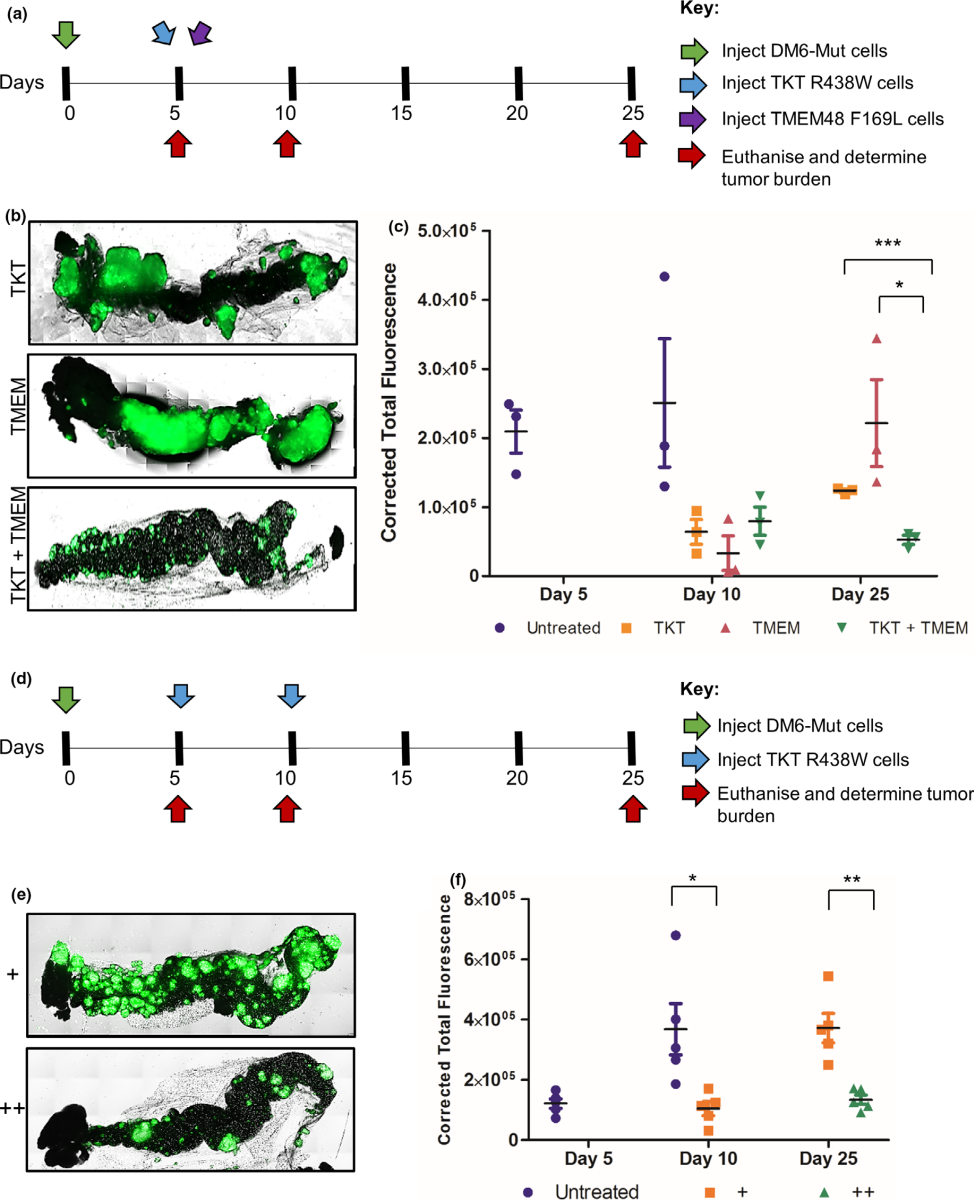
Tumor escape is suppressed by combining tumor‐specific T cells of different specificities or by repeated injections of T cells. **(a, d)** Experimental scheme indicating the timeline for injection of tumor cells (green arrow), TKT R438W cells (blue arrows), TMEM48 F169L cells (purple arrow) and estimation of tumor burden (red arrows). **(b, e)** Representative images of omental tumor burdens on day 25. **(c, f)** Tumor burdens are represented as corrected total fluorescence (CTF). *n* = 3 mice per group for **a–c** and 5 mice per group for **d–f**. There are no bars for untreated cohorts on day 25 as they were THTD. Data are presented as mean ± SEM. *P ≤ 0.05; **P ≤ 0.01, ***P ≤ 0.001. THTD, too high to determine accurately.

We first determined whether the adoptive transfer of a combination of two T‐cell populations derived from the same patient with specificity for two different tumor neoantigen peptides was therapeutically more effective than injecting the same number of T cells with a single peptide specificity. To address this, we introduced similar numbers of T cells with specificities for different tumor neoantigen peptides (TKT R438W and TMEM48 F169L) into DM6‐Mut xenografts individually or in combination (Figure 3a). We found that all three treated groups displayed early suppression of tumor growth on day 10 (compared to untreated). However, only the cohort that was given a combination of TKT R438W and TMEM48 F169L suppressed tumor recurrence, demonstrating a tumoristatic effect on day 25, while the TKT R438W as well as the TMEM48 F169L single treatment groups showed a 2‐ and 6.6‐fold increase in tumor burden, respectively (Figure 3b and c, and Supplementary figure 5a). We conclude from these results that combining T cells with different specificities is more efficacious and can significantly delay tumor escape in the X‐mouse model.

Next, we tested whether the therapeutic efficacy of the adoptive cell transfer was enhanced by repeating the T‐cell transfer. To assess the effect of reintroducing T cells in the xenograft, 1 × 10^6^ TKT R438W cells were introduced either on day 5 only (single treated) or on days 5 and 10 (double treated) into DM6‐Mut xenografts (Figure 3d). Reintroduction of TKT R438W cells on day 10 was found to significantly suppress the tumor escape seen in the single treated cohort (Figure 3e and f, and Supplementary figure 5b). These results are consistent with the loss of T‐cell function that has been reported to occur in tumor microenvironments, and establish repeated injections of T cells as a viable strategy to enhance anti‐tumor responses in the X‐mouse model.

Taken together, our results establish that the X‐mouse model as a reliable preclinical tool that can be used to test and optimise protocols for adoptive cell transfer therapies for cancer patients.

### Tumor escape mechanisms in the X‐mouse model

We next sought to identify and address mechanisms of tumor escape in the X‐mouse model. Of the multiple immunosuppressive factors in the tumor microenvironment, prevalent tumor escape mechanisms in patients include downregulation of MHC class I, loss of target peptides recognised by anti‐tumor T cells and checkpoint‐mediated downregulation of T‐cell responses in the tumor microenvironment.^22^ We did not find any evidence of MHC class I downregulation in DM6‐Mut xenografts (Supplementary figure 6a). Day 25 DM6‐Mut xenografts also expressed high levels of GFP, which is a reporter for cells bearing the TMC (and hence the peptide, since they are driven by the same promoter), which ruled out any significant loss (*P* = 0.662) of tumor antigen (Supplementary figure 6b).

Adoptively transferred T cells in the X‐mouse model may leave the omental xenografts and localise to other lymphoid organs/circulation over time. The detection of antigen‐specific CD8^+^ T cells in day 25 xenografts (Figure 1f) confirmed that these cells persist in the omentum for the duration of our experiments, ruling that out as a possible reason for tumor escape.

Persistently activated T cells have been reported to upregulate molecular markers of exhaustion, such as PD‐1, CTLA‐4, LAG3 and CD160,^22^ which correlate with their dysfunction. Based upon the assumption that the T cells in the xenograft are subject to persistent activation by the tumors, we predicted that the T cells would upregulate these checkpoint molecules, rendering them hyporesponsive to activation, and contribute to the tumor escape. Consistent with this possibility, we established that TKT R438W cells persistently activated with the cognate antigen *in vitro* upregulated the checkpoint molecules PD‐1, CTLA‐4, LAG3 and CD160 (Supplementary figure 7a). Furthermore, TKT R438W cells isolated from day 10 DM6‐Mut xenografts also showed high PD‐1 expression (Supplementary figure 7b). PD‐1 initiates immunosuppressive signalling by binding to its ligand, PD‐L1, that is expressed on multiple cell types in the tumor microenvironment including tumor cells,^23^ and is upregulated in response to IFN‐γ secreted by activated T cells.^24^ We found that the tumor target cells upregulated PD‐L1 when exposed to conditioned media derived from activated TKT R438W cells (Supplementary figure 7c). We also confirmed the expression of PD‐L1 in tumor xenografts by immunofluorescence labelling of omental wet mounts. PD‐L1 expression was first detected on day 15 (data not shown), and PD‐L1^+^ cells were seen in escaping tumors on day 25 (Supplementary figure 7d). No PD‐L1^+^ cells were detected in xenografts established using only DM6‐Mut cells (Supplementary figure 7d), indicating that T cells were required for PD‐L1 expression in the xenografts. While these results show the presence of PD‐L1 in DM6‐Mut tumor xenografts, a careful examination of these micrographs reveals that all tumor cells are not PD‐L1^+^, and does not rule out the possibility that PD‐L1 may be expressed on nontumor cells such as T cells in the tumor microenvironment. Collectively, these results provided a rationale for determining whether the upregulation of PD‐1 on the TKT R438W cells contributes to the tumor escape observed on day 25.

### Preclinical therapeutic efficacy of PD‐1 blockade demonstrated using the X‐mouse model

**Figure 4:**
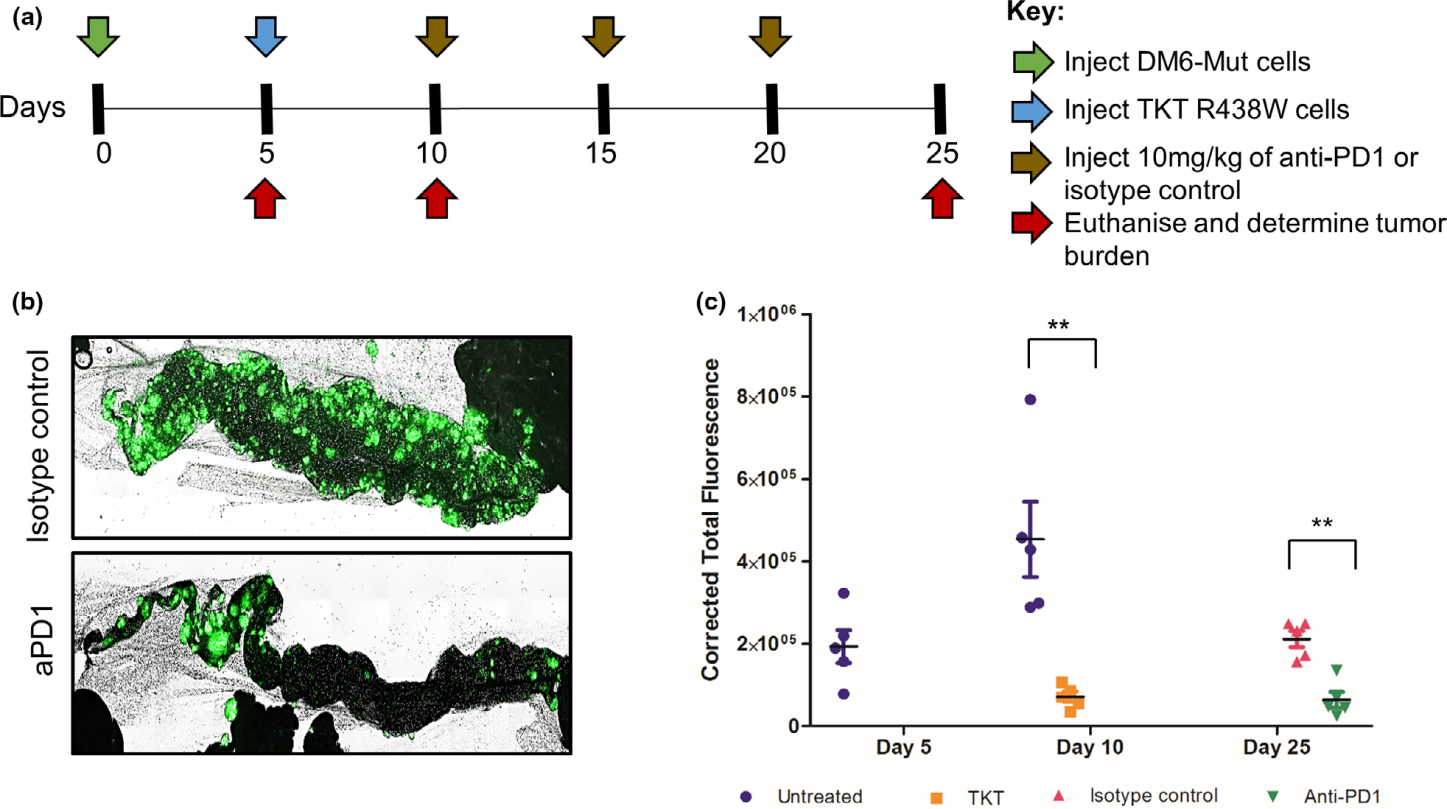
Anti‐PD1 treatment suppresses tumor escape in the X‐mouse model. **(a)** Experimental scheme indicating the timeline for injection of tumor cells (green arrow), TKT R438W cells (blue arrow), treatment with anti‐PD1 or isotype control (brown arrows) and estimation of tumor burden (red arrows). **(b)** Representative images of omental tumor burdens on day 25. **(c)** Tumor burdens are represented as corrected total fluorescence (CTF) on days 5, 10 and 25. There are no bars for untreated cohorts on day 25 as they were THTD. Since the mice that received TKT R438W cells on day 10 were divided equally and given either the isotype control or anti‐PD1, those are the only two cohorts on day 25. *n* = 5 mice per group. Data are presented as mean ± SEM. **P ≤ 0.01. THTD, too high to determine accurately.

The use of mAb to target the immunosuppressive PD‐1/PD‐L1 signalling (checkpoint blockade therapy) has been used successfully in the clinic, leading to multiple FDA‐approved biologics.^25^, ^26^ We tested the role of PD‐1 in tumor escape in the X‐mouse model by using nivolumab, an FDA‐approved anti‐PD1 mAb used in the treatment of metastatic melanoma.^27^ The antibody blockade of PD‐1 (Figure 4a) was shown to completely prevent tumor escape from T cell‐mediated suppression on day 25 (Figure 4b and c), providing confirmation that PD‐1 upregulation contributes to the observed tumor escape. More importantly, these results also provide the first validation of the X‐mouse model's ability to reflect the therapeutic efficacy of an established immune‐based therapy.

### Preclinical therapeutic efficacy of IL‐12 demonstrated using the X‐mouse model

**Figure 5:**
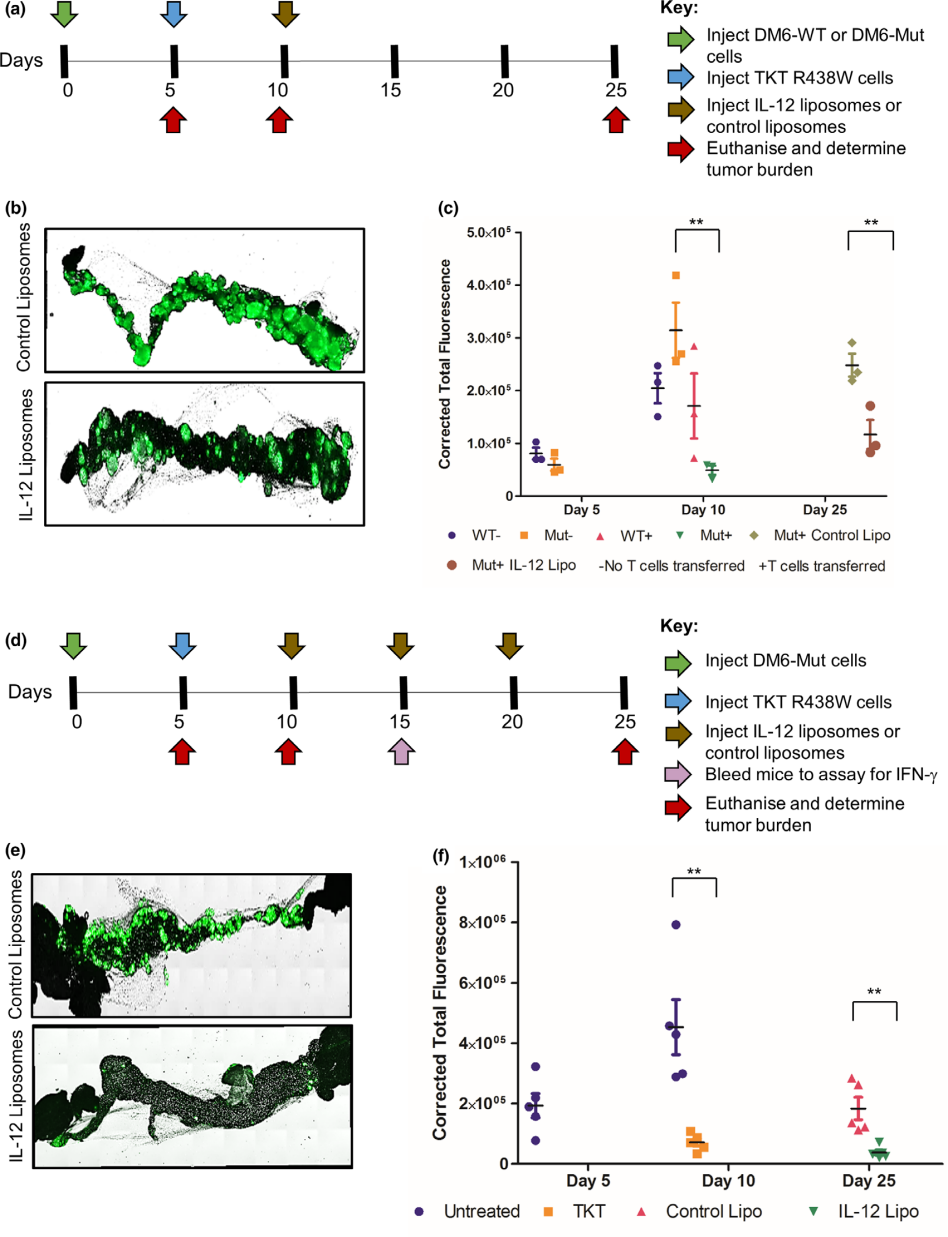
Liposomally delivered IL‐12 suppresses tumor escape in the X‐mouse model. IL‐12 was delivered once **(a–c)** or thrice **(d–f)** to DM6 xenografts. **(a, d)** Experimental scheme indicating the timeline for injection of tumor cells (green arrow), TKT R438W cells (blue arrow), treatment with IL‐12 liposomes or control liposomes (brown arrows) and estimation of circulating IFN‐γ (pink arrow) as well as tumor burden (red arrows). **(b, e)** Representative images of omental tumor burdens on the days 5, 10 and 25. **(c, f)** Tumor burdens are represented as corrected total fluorescence (CTF). *n* = 3 for **a–c** and 5 for **d–f**. There are no bars for untreated DM6‐Mut as well as DM6‐WT (untreated or treated) cohorts on day 25 as they were THTD. Since the mice that received TKT R438W cells on day 10 were divided equally and given either the empty (control) liposomes or the IL‐12 liposomes, those are the only two cohorts on day 25. Data are presented as mean ± SEM.**P ≤ 0.01. THTD, too high to determine accurately.

To further credential our model for evaluating immune‐based therapies, we next tested the ability of the model to mimic the anticipated efficacy of another therapy known to enhance T‐cell function in the tumor microenvironment, interleukin‐12. IL‐12 encapsulated in multilamellar liposomes has been found to activate T cells in tumor microenvironments established in PDX.^11^ We predicted that introduction of IL‐12 into the X‐mouse xenografts would result in an activation of the T cells that would ultimately result in the prevention or delay of the tumor escape. To test this prediction, 20 µg of IL‐12 encapsulated into multilamellar liposomes was injected i.p. into xenograft‐bearing mice (Figure 5a). Control groups were treated with empty liposomes. We found that there was a partial suppression of DM6‐Mut tumor escape on day 25 in the IL‐12‐treated cohort (Figure 5b and c, and Supplementary figure 8a), confirming the viability of this approach. IL‐12 had no effect on tumor burden in DM6‐WT xenografts treated with TKT R438W cells, ruling out nonspecific effects of either IL‐12, or IL‐12‐activated TKT R438W cells on control tumor cells.

We next attempted to enhance the observed therapeutic effect of IL‐12 liposomes by their repeated administration and determine whether the predicted enhancement of the anti‐tumor response would be reflected in the X‐mouse model. For this, the experiment was designed similarly, but with additional liposomal IL‐12 injections on days 15 and 20 (Figure 5d). DM6‐WT controls were not used since we had already demonstrated the absence of any detectable nonspecific effects. We found that by modifying the schedule, a greater suppression of the tumor was reflected in the model. This treatment schedule not only prevented tumor escape, but also knocked down the tumor burden from day 10 by 50% (Figure 5e and f). Because IL‐12‐mediated stimulation of T cells leads to expression of IFN‐γ, we tested circulating levels of IFN‐γ on day 15 (i.e. 5 days after the first injection of IL‐12) by ELISA.^28^ Indeed, the mean IFN‐γ levels were found to be threefold higher in the cohort that received IL‐12 liposomes compared to the one that received control liposomes, consistent with enhanced T‐cell stimulation as a result of IL‐12 injection (Supplementary figure 8b). Together, these results validate our model for its ability to mimic anticipated results of another immune‐based therapy.

### Monitoring the effect of immunotherapy on patient‐derived anti‐tumor T cells in the tumor microenvironment using the X‐mouse model

**Figure 6:**
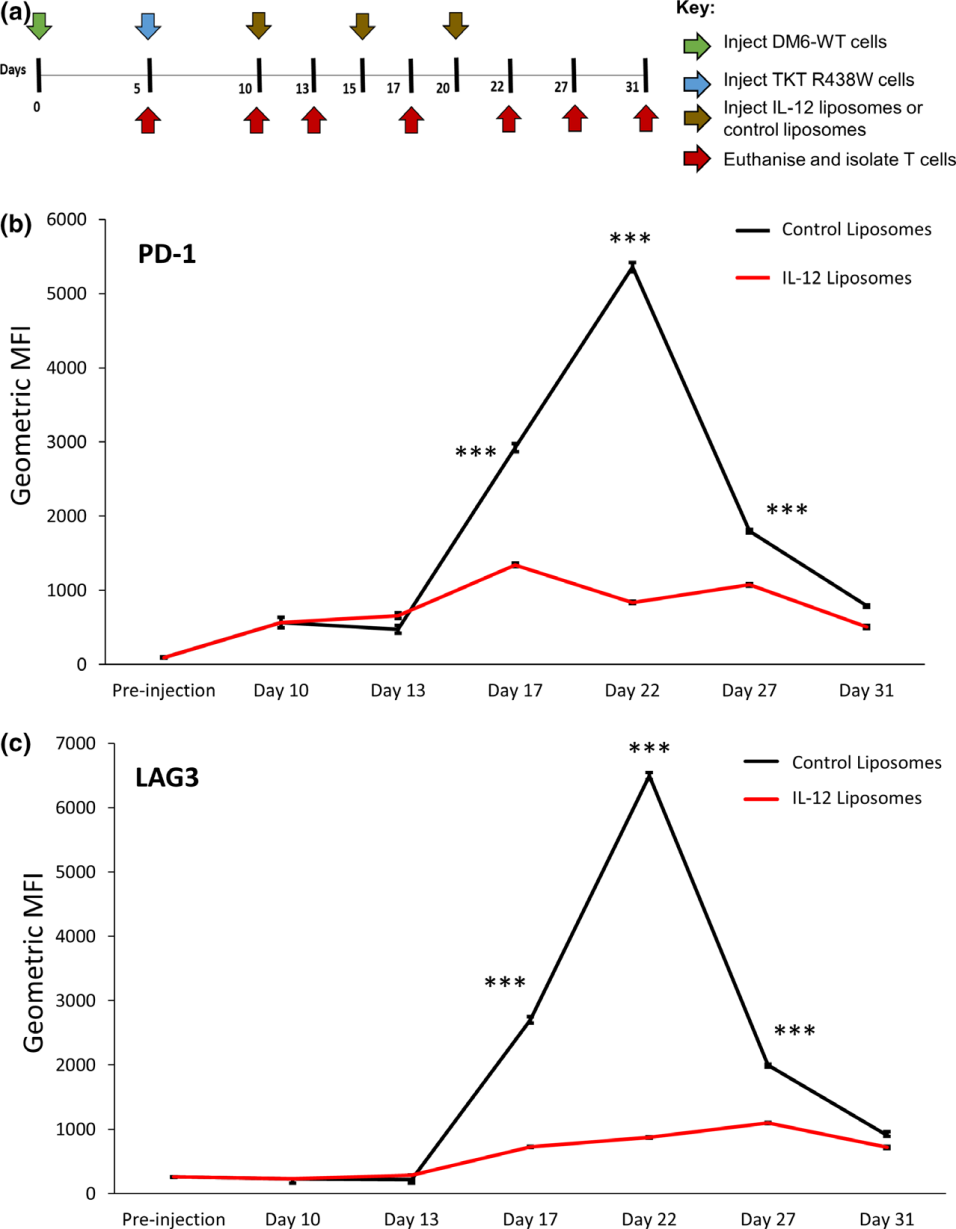
IL‐12 suppresses the upregulation of checkpoint molecules in TKT R438W cells. **(a)** Experimental scheme indicating the timeline for injection of tumor cells (green arrow), TKT R438W cells (blue arrow), treatment with IL‐12 liposomes or control liposomes (brown arrows) and estimation of tumor burden (red arrows). Kinetics of PD‐1 **(b)** and LAG3 **(c)** expression in TKT R438W cells following their entry into DM6‐Mut tumor xenografts in different experimental cohorts are shown. Mice were euthanised on the indicated days, and T cells derived from the omenta were analysed by flow cytometry. Gated on CD3^+^ cells as shown in Supplementary figure 9. Data (*n* = 3 mice per group) represented as mean ± SEM. ***P ≤ 0.001.

*Ex vivo* exposure to IL‐12 has been shown to protect tumor‐infiltrating murine CD8^+^ T cells from negative regulation by IFN‐γ via downregulation of IFNγR2 and PD‐1 expression in a mouse model of melanoma.^29^ Additionally, intratumoral electroporation with IL‐12 has been shown to result in a coordinated downregulation of multiple checkpoints including PD‐1 and LAG3 in murine CD8^+^ T cells.^30^ We therefore tested whether IL‐12 treatment affected the expression of the checkpoint molecules PD‐1 and LAG3 on patient‐derived anti‐tumor T cells in the X‐mouse model. The xenografts for the different experimental cohorts were established as described (Figure 6a), and T cells were recovered from the xenografts by the previously described walk out method^31^ at multiple time points. The expression of PD‐1 and LAG3 on T cells (CD3^+^ cells gated as shown in Supplementary figure 9) was determined by flow cytometry. Following their entry into the DM6‐Mut xenograft, PD‐1 expression on TKT R438W cells was initially upregulated by about sixfold on day 10 (Figure 6b), that is 5 days following T‐cell injection, as observed earlier (Supplementary figure 7b). These levels continued to increase, peaking at day 22 with over 55‐fold increase over pre‐injection levels (Figure 6b). Injection of IL‐12 liposomes suppressed this upregulation at all the time points studied (Figure 6b). LAG3 expression followed slightly different kinetics, with upregulation seen only at time points beyond day 13, while also peaking at day 22 (Figure 6c). As observed in the case of PD‐1, IL‐12 was found to suppress the upregulation of LAG3 at all the time points studied (Figure 6c). We show here for the first time, the ability of IL‐12 immunotherapy to suppress the upregulation of checkpoint molecules in patient‐derived anti‐tumor T cells in the tumor microenvironment.

Taken together, these data establish that the X‐mouse model is a powerful preclinical tool for rapidly evaluating immunotherapy protocols by monitoring changes in the tumor burden as well as in the phenotype of tumor‐specific T cells at the cellular level.

## Discussion

As the first tumor xenograft model to provide a controlled system in which the number of tumor cells used to establish tumor xenografts is known, the timing and number of T cells that are adoptively transferred into tumor‐bearing mice are controlled, and the tumor specificity of the T cells for tumor antigens is well‐defined, the X‐mouse model offers several advantages over existing PDX models. The DM6 cell line was selected for our study as it is an established melanoma model for immunotherapy with high clinical relevance. Additionally, the HLA type of DM6 cells was compatible with that of the patients in the neoantigen vaccination trial. This ensures that once the cells are transduced with cells with TMC with the mutated tumor peptides, these peptides are processed and presented in the context of MHC‐I on their surface, turning the DM6‐Mut cells into target cells that are recognised and killed by the patient's tumor‐specific T cells. With this approach, we have established the presence of multiple tumor antigen‐specific T cells in three different vaccinated patients. We have also demonstrated the utility of our model using melanoma cells expressing two different patient tumor‐derived neoantigen peptides (TKT R438W and TMEM48 F169L) and the T cells that recognise these peptides. The use of DM6‐WT tumors, where the tumor cells express the nonmutated/wild‐type peptide which differs from the neoantigen peptide by only one amino acid, provides an elegant control, and the failure of the adoptively transferred T cells to suppress these tumors establishes the specificity of the anti‐tumor response in our model. High T‐cell yields following expansion (3.6–10.8 × 10^8^ cells per neoantigen, Supplementary figure 1b), coupled with relatively low T‐cell number requirements to establish an X‐mouse xenograft (0.5–1 × 10^6^), translate into at least 300–1000 xenografts per antigen‐specific T‐cell population. The X‐mouse model has made it possible to obtain and quantify at intervals, changes in the number, phenotype and function of the T cells in the tumor microenvironment with and without therapy, as well as to demonstrate the ability of the tumor to escape the T‐cell response and metastasise, thereby providing a distinct advantage over simple *in vitro* co‐culture systems for preclinically evaluating the efficacy of immune‐based therapies.

Our use of GFP and/or luciferase represents an accurate and highly sensitive way for quantifying tumor burden in the X‐mouse model, providing advantages over existing methods such as measurement of tumor volume using callipers, which does not distinguish between tumor, stromal and immune cells, in addition to being error prone^32^; and the detection of tumor transcripts,^33^ which involves multiple processing steps that can be time‐consuming as well as labour‐intensive. GFP allows rapid measurement of tumor burden *post‐mortem* by scanning wet mounts of the omental tissue directly under the fluorescent microscope without any need for processing or immunofluorescence labelling. While using GFP as a marker of tumor burden does have some limitations such as its relatively low permeability through tissue—a problem that is aggravated by stromal thickening during tumor growth—this is considerably offset in the X‐mouse model because of the translucent nature of the omentum, which is the primary site of the xenograft. The sensitivity of the X‐mouse model can be improved upon in future iterations by replacing GFP with red and far‐red‐emitting proteins that have better tissue penetration and consequently lesser loss of signal. While end‐stage GFP quantification is lacking in this study because of high tumor burden in the omenta which makes the signal THTD, further optimisation of exposure times to enable the measurement of GFP signal over a wider spectrum is expected to overcome this. Luciferase is useful for *in vivo* monitoring of the tumor burden and particularly to track metastasis.^34^ Further, it may be used for *ex vivo* monitoring of omental tumor burden at the terminal time point, providing another means to overcome the lack of end‐stage tumor quantification.

The rapid and preferential localisation of both the melanoma tumor target cells and the tumor‐specific T cells into the greater omentum in our model has made it possible to recover and quantify changes at the cellular level in the number and phenotype of the tumor cells within the xenograft and in the number, phenotype and function of the anti‐tumor T cells following their entry into the tumor microenvironment. While we recognise that the omentum is not a natural or preferential site of metastasis for melanomas in patients, ovarian tumors naturally metastasise to the omentum in patients,^35^, ^36^ and many different types of human tumors engraft rapidly and preferentially in the greater omentum of mice,^9^, ^21^ making it an ideal anatomical location for establishing human tumor xenografts. Another unique and significant advantage of the X‐mouse is the rapid engraftment of tumors that have made it possible to generate results within just 25 days for post‐mortem studies and tumor progression but can potentially be monitored continuously for longer periods using live imaging of mice.

Although adoptive cell therapy has made huge strides in cancer treatment, especially with the emergence of CAR T cells, tumor relapse/recurrence is common^37^ and remains a challenge. The escape of DM6‐Mut tumors following the initial adoptive transfer of tumor‐specific T cells is critical to our model as it allows us to test immune‐based therapies designed to enhance anti‐tumor T‐cell activity of adoptively transferred T cells by monitoring their ability to prevent or delay the tumor escape. Our discovery of the involvement of the PD‐1/PD‐L1 axis in the X‐mouse xenografts allowed us to test two immune‐based therapies that target this immunosuppressive mechanism. First, we demonstrated that checkpoint blockade using nivolumab, an FDA‐approved anti‐PD‐1 antibody that is currently used in the clinic, was able to suppress tumor escape in the X‐mouse model, validating our model for immunotherapeutic testing. The second immune‐based therapy that we tested, IL‐12, is predicted to enhance anti‐tumor T‐cell response by two mechanisms: stimulating T cells directly through its receptor IL‐12R and by protecting T cells from IFN‐γ mediated negative regulation by downregulating checkpoint expression. As expected, not only did treatment with IL‐12 result in enhanced anti‐tumor response as seen by the suppression of tumor escape, it also resulted in the suppression of PD‐1 and LAG3 upregulation, providing further credentialing of the X‐mouse model. The ability to monitor checkpoint kinetics in response to immune therapies in patient‐derived tumor‐specific T cells is a unique and unmatched feature of the X‐mouse model. We thus establish the potential of the model to reflect/mimic the predicted enhanced anti‐tumor responses of the adoptively transferred T cells in response to immune‐based therapies.

One of the major characteristics that set the X‐mouse model apart from other PDX models is that the adoptively transferred T cells are the only immune competent cells in the xenograft. While this may be considered a limitation for certain applications, it also provides us with a distinct advantage. Enhanced anti‐tumor activity seen as a response to any immunotherapy being tested in the X‐mouse model will be a result of directly affecting the dynamic between tumor cells and T cells, making the interpretation of results far less complicated than it would be in the presence of other immune cells in the microenvironment. Therefore, our model is best suited to test immunotherapies that are designed to enhance T‐cell function and is expected to aid in rapid go/no‐go decisions at the preclinical stage of development.

While we establish the feasibility of the X‐mouse model here using DM6 cells, we recognise the need to include more tumor cell lines to demonstrate the robustness of our model and its broad applicability. The use of a single tumor cell line also has the additional limitation of not being able to recapitulate the diversity of mutational profiles that occur in tumors from different patients. We plan to overcome this by generating additional tumor target cells derived from the patient's tumor in the future. We also plan to investigate the role of additional immune checkpoints such as other exhaustion markers (CTLA‐4, Tim‐3 and CD160), tumor‐associated exosomes^38^, ^39^, ^40^ as well as exosome‐associated immunosuppressive lipids such as phosphatidylserine^41^ and ganglioside GD3^42^, ^43^ in the observed tumor escape in future studies. The efficacy of checkpoint blockade can also be enhanced many fold with combination therapy, which can be tested rapidly in our model. As the only existing model that can preclinically evaluate the efficacy of immune‐based therapies using patient‐derived tumor‐specific T cells, we believe that harnessing the potential of the X‐mouse model can bridge the gap between preclinical promise and clinical success.

## Methods

### Neoantigen‐specific T‐cell generation

Patients with stage III cutaneous resected melanoma were enrolled in a phase 1 clinical trial with autologous, functionally mature, interleukin‐12p70 (IL‐12p70)–producing dendritic cell vaccine. Exome sequencing was performed to identify somatic mutations in surgically excised tumor samples. Tumor missense mutations, translated as amino acid substituted nonamer peptides, were filtered through *in silico* analysis to determine HLA‐A*02:01 peptide‐binding affinity. Selected peptides from validated HLA‐A*02:01 binders were incorporated into a personalised vaccine formulation. Neoantigen‐specific CD8^+^ T cells were identified by dextramer assay directly in postvaccine PBMC samples. CD8^+^ T cells specific for two neoantigens—TKT R438W (TKT) and TMEM48 F169L (TMEM) in patient MEL21—were expanded from leukapheresis products and used for this study (Supplementary figure 1b).

### Tumor target cell generation

DM6, an HLA‐A*02:01^+^ melanoma cell line, was transfected with TMCs and serves as a target for *in vivo* presentation of neoantigens, TKT R438W and TMEM48 F169L or wild‐type counterparts. TMC consisting of 7–10 minigenes cloned into pMX vector (GFP^+^), expressed as retrovirus and used to transduce the HLA‐A*02:01^+^ melanoma line DM6. The TMC as well as the GFP is driven by the cytomegalovirus immediate early promoter. Each minigene encoded an amino acid substituted, or the corresponding wild‐type amino acid, embedded in 19–21 amino acids derived from the normal gene product. Both TMEM48 F169L and TKT R438W were processed and presented, as evidenced by cytotoxic activity and IFN‐γ production by corresponding neoantigen‐specific T cells upon co‐culture with DM6 cells expressing mutated neoantigens (DM6‐Mut) but not wild‐type‐encoding TMCs (DM6‐WT). DM6‐WT and DM6‐Mut luciferase‐expressing cells (DM6‐WT‐LUC^+^ and DM6‐Mut‐LUC^+^) were generated by retroviral transduction followed by neomycin selection.

### Cell culture

DM6‐Mut and DM6‐WT cells were maintained in RPMI‐1640 supplemented with 10% heat‐inactivated FBS, 2 mm l‐glutamine, 20 U mL^−1^ penicillin, 20 µg of streptomycin and 50 µm 2‐mercaptoethanol (complete medium) at 37°C under 5% CO_2_. Cells are adherent and were grown in 75‐ or 175‐cm^3^ flasks until they were 90% confluent. For cell harvesting, cultures are treated with 0.25% trypsin–EDTA for 3–4 min at 37°C following removal of the medium and one wash with phosphate‐buffered saline (PBS). Complete medium is added to stop the reaction and cells are centrifuged at 300 *g* for 10 min.

### Authentication of cell lines

The parental DM6 cell line was obtained from Dr Hilliard Seigler at Duke University. The identity of the cell line has been confirmed by concordance between reported and tested HLA class I typing. DM6 cells were molecularly HLA typed using next‐generation sequencing and assignments of HLA‐A and HLA‐B alleles corroborated those reported in the Expasy database (www.expasy.org). DM6 cells were transduced with retroviruses encoding the TMC, Rho C and click beetle green luciferase and/or green fluorescent protein (GFP). These cells were selected based on GFP expression (> 99% GFP‐positive) and expanded. Cells were cultured in complete DMEM media and periodically monitored for transgene expression. Banks of the above‐engineered DM6 cells were also screened and confirmed to be free of mycoplasma contamination. The cells from any one cryopreserved vial were not passaged for more than 2 months after being thawed out. These cells were tested for GFP expression and confirmed to be > 99% positive on the day of the experiments.

### Mice

NOD.Cg‐*Prkdc^scid^ Il2rg^tm1Wjl^*, abbreviated NSG mice (young adult females 8–12 weeks old), raised in a research colony at the Jackson Laboratory (Bar Harbor, ME, USA), were used for this study. The sample size for *in vivo* experiments was calculated based on the ability to demonstrate with 85% power, a 2‐fold difference in tumor burden, allowing for a 30% standard deviation in tumor size and spread assuming a normal distribution of the samples. Age‐ and sex‐matched mice were assigned to control and test groups.

### Establishment of xenografts

NSG mice were implanted with 2.5 × 10^6^ DM6‐Mut or DM6‐WT cells i.p. in a total volume of 0.5 mL. Cryopreserved neoantigen‐specific TKT R438W or TMEM48 F169L cells were thawed and incubated in complete medium for 6 h at 37°C and 5% CO_2_. Unless noted otherwise, 5 × 10^5^ or 1 × 10^6^ T cells were injected i.p. per mouse in a total volume of 0.5 mL, 5 days following the implantation of DM6 cells. While the experiments demonstrating the *in vivo* efficacy of anti‐PD1 (Figure 4) and IL‐12 (with three treatments, Figure 5d–f) were set up at the same time and therefore shared day 5 and 10 controls, all other xenografts were set up independently. All experiments were independently repeated at least once, and data from a representative experiment are shown. All animal studies were approved by the Institutional Animal Care and Use Committee.

### Post‐mortem analysis of tumor burden

Twenty‐five days following tumor implantation, the mice were euthanised and the greater omentum from each mouse was surgically removed. A wet mount of the omentum in PBS was scanned using the Leica DM6 B upright fluorescence microscope (Leica Microsystems, Wetzlar, Germany). The entire omentum was scanned under the 5× objective using the GFP and DIC filters. The images were exported as TIF files and analysed using ImageJ software (National Institutes of Health, Bethesda, MD, USA) to quantify the GFP signal. The polygon tool was used to draw a tight border around the omentum, and the amount of signal was measured in the green channel. Background in the green channel was also measured by drawing a gate in a region of the omentum that was free of any tumor cells. Corrected total fluorescence was then calculated using the formula CTF = intensity density of omentum − (area of omentum × mean grey value of background for that omentum).

### *In vivo* analysis of tumor burden

Tumor cells were implanted in the omentum as described above. Local and disseminated tumor burden was monitored by bioluminescent imaging. Mice were anaesthetised with 4% isoflurane and injected i.p. with substrate d‐luciferin (Gold BioTechnology #LUCK‐1G, St. Louis, MO, USA) at 150 mg kg^−1^ in Dulbecco's PBS and, after a 5‐min interval, were placed onto the warmed stage inside the light‐tight camera box of the imager (IVIS™ Spectrum; Perkin Elmer, Waltham, MA, USA) while under 2% isoflurane. Light emitted from bioluminescent cells was recorded by the IVIS® camera system. Images were quantified for tumor burden using a log‐scale colour range, and measurement of total photon counts per second (p/s) was determined using Living Image software (Perkin Elmer).

### Recovery of T cells from xenografts

T cells were recovered from the omenta of xenograft‐bearing mice by the previously described walkout method.^31^ Omenta were harvested under sterile conditions and placed in complete medium in wells of a 6‐well tissue culture plate. The omenta were gently cut into small 4‐ to 5‐mm pieces and incubated overnight at 37°C under 5% CO_2_. On the following day, the medium with the ‘walkout cells’ was passed through a 70‐µm cell strainer to exclude omental tissue and debris.

### Histology

Fresh tissue was fixed in 10% neutral‐buffered formalin and processed for paraffin embedding. SUNY Buffalo Histology Service Laboratory performed the H&E staining of the omental tissue.

### Checkpoint blockade therapy

10 mg kg^–1^ of nivolumab (OPDIVO), a humanised anti‐PD1 antibody (Bristol‐Myers Squibb, East Syracuse, NJ, USA), or human IgG4 isotype control (Crown Bioscience, San Diego, CA, USA), was administered i.p. in a total volume of 200 µL per mouse on days 10, 15 and 20.

### Preparation and administration of IL‐12 liposomes

IL‐12 liposomes were prepared as previously described.^10^ Large multilamellar liposomes were prepared by rehydrating the lipid film of appropriate molar ratios of distearoyl phosphatidylcholine (DSPC), dimyristoyl phosphatidylglycerol (DMPG) and cholesterol (CHOL) (DSPC∶DMPG∶CHOL; 50∶50∶25) with phosphate buffer containing recombinant human IL‐12 at 45°C. The spontaneous loading of a large complex molecule such as IL‐12 is accomplished by a mild denaturation of IL‐12 to partially and reversibly unfold the protein to expose hydrophobic domains resulting in the intercalation of the cytokine within or between the lipid bilayers of the liposomes. This technique called triggered loading results in an optimal loading of the IL‐12 which retains its biological activity. After rehydration, lipids are gently swirled and incubated at 45° for 20 min. 20 µg of IL‐12 liposomes or empty (control) liposomes was administered to mice i.p. in a total volume of 500 µL on day 10.

### Flow cytometry

Immunofluorescence labelling was performed as described previously^38^ using fluorochrome‐conjugated antibodies and reagents are listed in Supplementary table 2. Briefly, 1 × 10^6^ cells were set up in each tube in PBS for immunofluorescence labelling. The cells were blocked with normal mouse IgG for 10 min, and directly labelled antibodies were added and incubated on ice for 30 min. In experiments where MHC Dextramer reagents were used to detect antigen‐specific T cells, they were added to the tubes 10 min prior to the addition of the antibodies. Sytox Red was added 15 min before flow cytometry at a final concentration of 5 nm to label the dead cells. Samples were acquired on an LSR Fortessa (BD Biosciences, San Jose, CA, USA) flow cytometer. A minimum of 2 × 10^4^ lymphocytes were acquired for analysis of T cells from xenografts, and for other studies, data acquisition was stopped after acquiring 5 × 10^4^ lymphocytes. Data analysis was performed using FlowJo software (Tree Star Inc., Ashland, OR, USA).

### PD‐L1 expression in conditioned media

DM6‐Mut cells were plated onto 6‐well plates and grown to 60–80% confluency for 48 h. TKT R438W T cells were then added in fresh media at a ratio of 1:1.5 (tumor cell:T cell) and incubated for an additional 48 h. Media was collected, and cells and debris were pelleted by centrifugation at 1150 x *g* for 5 min. Supernatant was collected and added at varying dilutions to new DM6‐Mut cells grown to ~50% confluency. These T‐cell conditioned media‐treated DM6‐Mut cells were grown for an additional 48 h before protein was collected and probed for PD‐L1 expression by western blot.

### Western blots

Cell protein was harvested using RIPA buffer (G Biosciences, St. Louis, MO, USA) supplemented with Halt protease inhibitor (Thermo Fisher Scientific, Grand Island, NY, USA). Protein concentration was measured using the DC protein assay (Bio‐Rad, Hercules, CA, USA). Electrophoretic separation of protein (12–20 μg/well) was performed using 4–15% gradient polyacrylamide gels (Bio‐Rad). Separated protein was transferred onto PVDF membranes (Bio‐Rad), which were then blocked for 1 h at room temperature in Tris‐buffered saline containing 0.1% tween (TBS‐T) with 5% fat‐free milk (Bio‐Rad), followed by overnight incubation at 4°C with rabbit anti‐human PD‐L1 antibody (Cell Signaling Technologies, #13684T, Danvers, MA, USA) (1:1000 dilution) or mouse anti‐human β‐actin antibody (1:10 000 dilution) (Cell Signaling Technology, #3700 S) in 5% fat‐free milk with TBS‐T. Membranes were washed in TBS‐T and incubated for 20 min at room temperature with a 1:2000 dilution of horseradish peroxidase‐conjugated goat anti‐rabbit antibody (Promega, #W4011, Madison, WI, USA) or rabbit anti‐mouse antibody (Cell Signaling Technology, #7076 S) in 5% milk with TBS‐T. Protein signals were developed using the WesternBright ECL HRP substrate (Advansta, #K‐12045, San Jose, CA, USA) and measured using a Chemi‐Doc MP scanner (Bio‐Rad).

### *In vivo* detection of PD‐L1 by immunofluorescence labelling of omental whole mounts

The greater omentum was surgically removed and transferred to polypropylene tubes containing PBS. For blocking nonspecific binding, normal mouse IgG was added and incubated for 10 min with rocking. Brilliant Violet 421‐labelled anti‐PD‐L1 (BioLegend, #329714, San Diego, CA, USA) was added and incubated for 2 h with rocking at 4°C. Samples were washed twice in PBS for 15 min each and mounted on a slide. Images were acquired using a Leica DM6 B upright fluorescence microscope (Leica Microsystems, Wetzlar, Germany) and analysed using ImageJ software.

### Human Interferon Gamma ELISA

A sandwich ELISA for the detection of human IFN‐γ in mouse serum was performed as previously described.^28^ Briefly, 96‐well plates were coated using a mAb to IFN‐γ (ThermoFisher Scientific, #M‐700‐A, Grand Island, NY, USA). Mouse sera were added to the plate coated with biotinylated monoclonal anti‐human IFN‐γ (ThermoFisher Scientific, #M‐700‐B). Positive binding was detected using streptavidin‐conjugated HRP (Sigma‐Aldrich, #A3151, St. Louis, MO, USA), peroxide and 3,3′,5,5′‐tetramethylbenzidine (Kirkegaard & Perry Laboratories, #52‐00‐01, Gaithersburg, MD, USA). Results were measured on a MultiSkan EX automated microplate reader (Thermo Electron Corporation, Waltham, MA, USA) at OD450–540 and analysed by comparison with a recombinant IFN‐γ standard using SigmaPlot software (Systat Software Inc., San Jose, CA, USA).

### Statistics

All statistics were calculated using GraphPad (GraphPad Software, San Diego, CA) or Excel 2013 (Microsoft Corporation, Redmond, WA). Paired or unpaired Student's *t*‐tests were applied to determine whether the differences between groups could be considered significant. A *P*‐value higher than 0.05 was not significant (ns) while *P ≤ 0.05; *P ≤ 0.01 and ***P ≤ 0.001 were considered significant.

### Study approval

The study was approved by the Institutional Review Board (protocol MODCR00004766), and all animal experiments were approved by the Institutional Animal Care and Use Committee (protocol MIC17072Y) at the University at Buffalo.

## Conflict of interest

Triggered loading technology used in this study has been patented (US Patent 7 662 405 entitled ‘Composition and Method of Liposomal Microparticulate IL12’), and Immune Modulatory Therapies, LLC, has exclusive rights to this patent. The authors declare no other conflicts of interest.

## Author contributions

**Gautam Nitin Shenoy:** Conceptualization; Investigation; Methodology; Writing‐original draft; Writing‐review & editing. **Christopher J Greene:** Investigation; Methodology; Writing‐review & editing. **Maulasri Bhatta:** Investigation; Methodology; Writing‐original draft. **Miren L Baroja:** Investigation. **Jenni L Loyall:** Investigation; Methodology; Project administration. **Sathy V Balu‐Iyer:** Funding acquisition; Methodology; Resources; Supervision. **Raymond J Kelleher Jr:** Resources; Supervision; Writing‐review & editing. **Beatriz M Carreno:** Funding acquisition; Investigation; Methodology; Resources; Supervision; Writing‐review & editing. **Gerald Linette:** Funding acquisition; Resources; Supervision; Writing‐review & editing. **Leonard D Shultz:** Funding acquisition; Resources; Supervision; Writing‐review & editing. **Richard B Bankert:** Conceptualization; Funding acquisition; Resources; Supervision; Writing‐original draft.

## Data Availability

Data are available upon reasonable request. All data relevant to the study are included in the article or uploaded as Supporting information Any questions regarding published results, reagents or method should be directed to the corresponding author Dr Richard B Bankert.
