# Supplementary material for: Preclinical evaluation of cancer immune therapy using patient‐derived tumor antigen‐specific T cells in a novel xenograft platform
Source: Clin Transl Immunology. 2021 Feb 2;10(2):e1246. doi: 10.1002/cti2.1246 (PMC7853904; doi:10.1002/cti2.1246)
Supplement: Supplementary file 1 [file CTI2-10-e1246-s001.pdf]

(a)

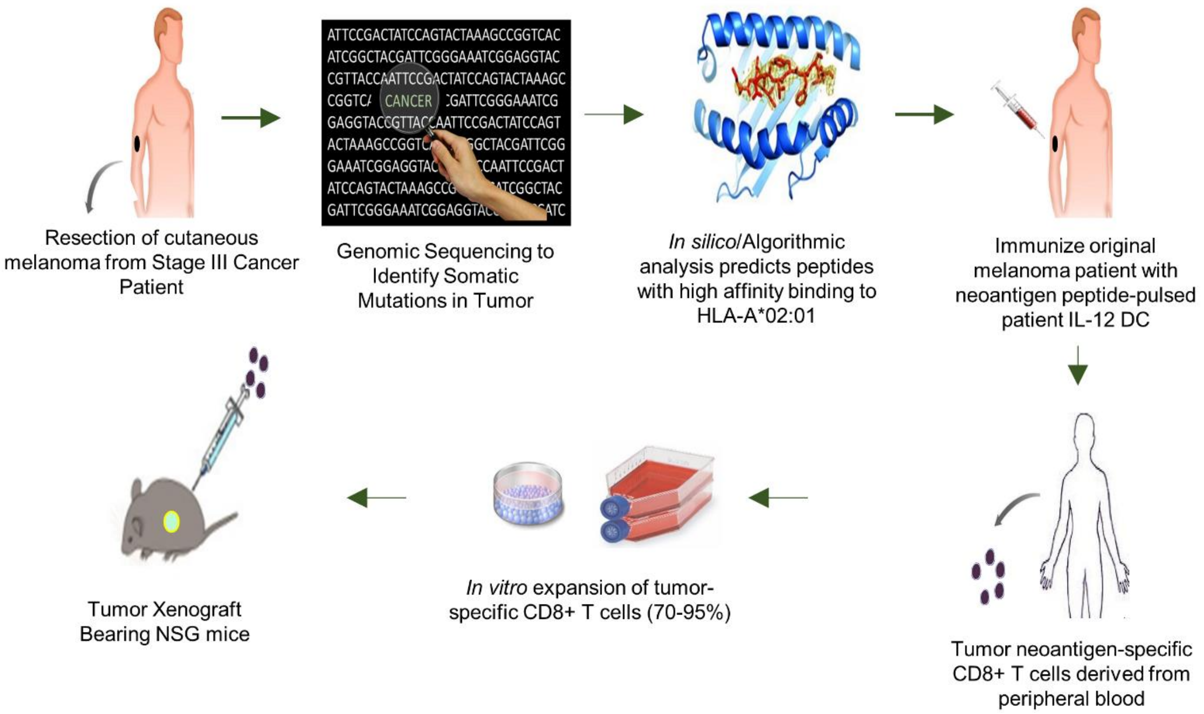

(b)

Patient	T cells	Mutated Peptide Recognized	Starting cell number x 10^6	After primary expansion x 10^6	After secondary expansion x 10^8
Mel 21	TKT R438W	AMFWSVPTV	2.5	15.8	10.8
	TMEM48 F169L	CLNEYHLFL	1	13.8	3.66

Supplementary Figure 1: Generation of tumor neoantigen-specific T cells. **(a)** Outline of the approach that is taken to generate and expand neoantigen specific T cells derived from melanoma patients vaccinated with IL-12 producing DC loaded with the tumor neo-antigen peptide. **(b)** Details and yields of antigen-specific T cells that are expanded. Peptide sequences for each neoantigen are listed and mutated amino acid is underlined.

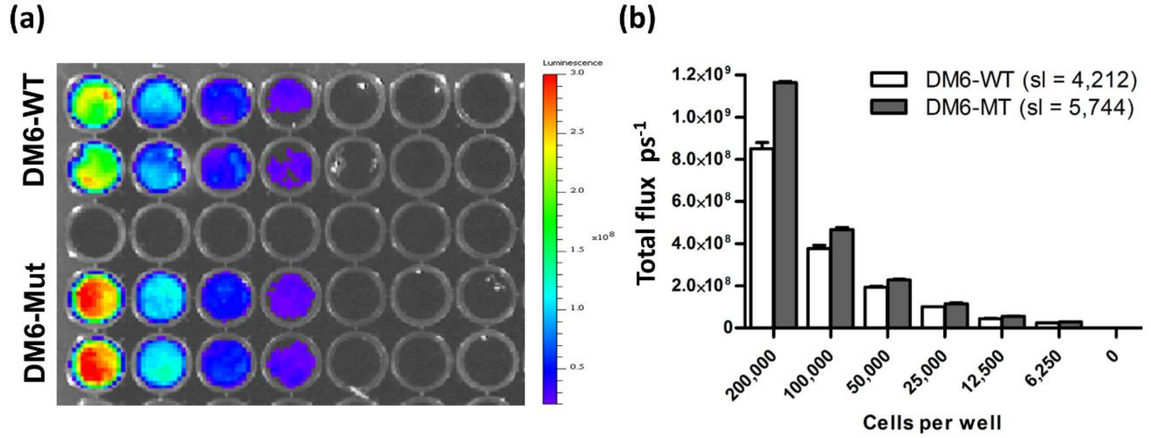

Supplementary Figure 2. Luciferase expression in DM6-WT and DM6-Mut cells. DM6-WT or DM6-Mut cells transduced with luciferase were serially diluted, co-incubated with luciferin and imaged. **(a)** Representation of photon flux in each group **(b)** Quantified photon flux depicted as Mean ± SD

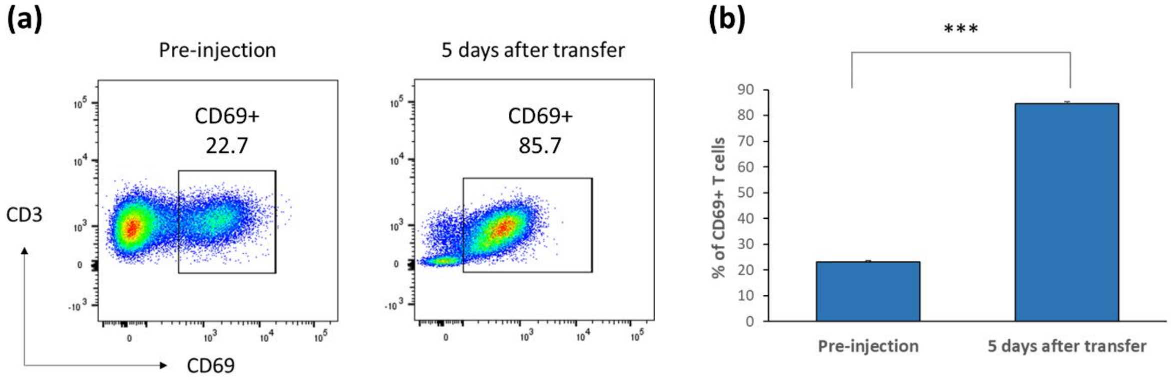

Supplementary Figure 3. Adoptively transferred T cells are activated following entry into DM6-Mut xenografts**.** 5 x 10^5 TKT R438W cells were adoptively transferred into 5-day old xenografts established by i.p. injection of 2.5 x 10^6 DM6-Mut cells into NSG mice. The T cells were isolated from the xenografts 5 days after their transfer (i.e. experimental day 10) and the expression of CD69 on live T cells was determined by flow cytometry. Representative data shown in **(a)** and quantified data shown in **(b)**. $n = 3$. $^{***}P \leq 0.001$

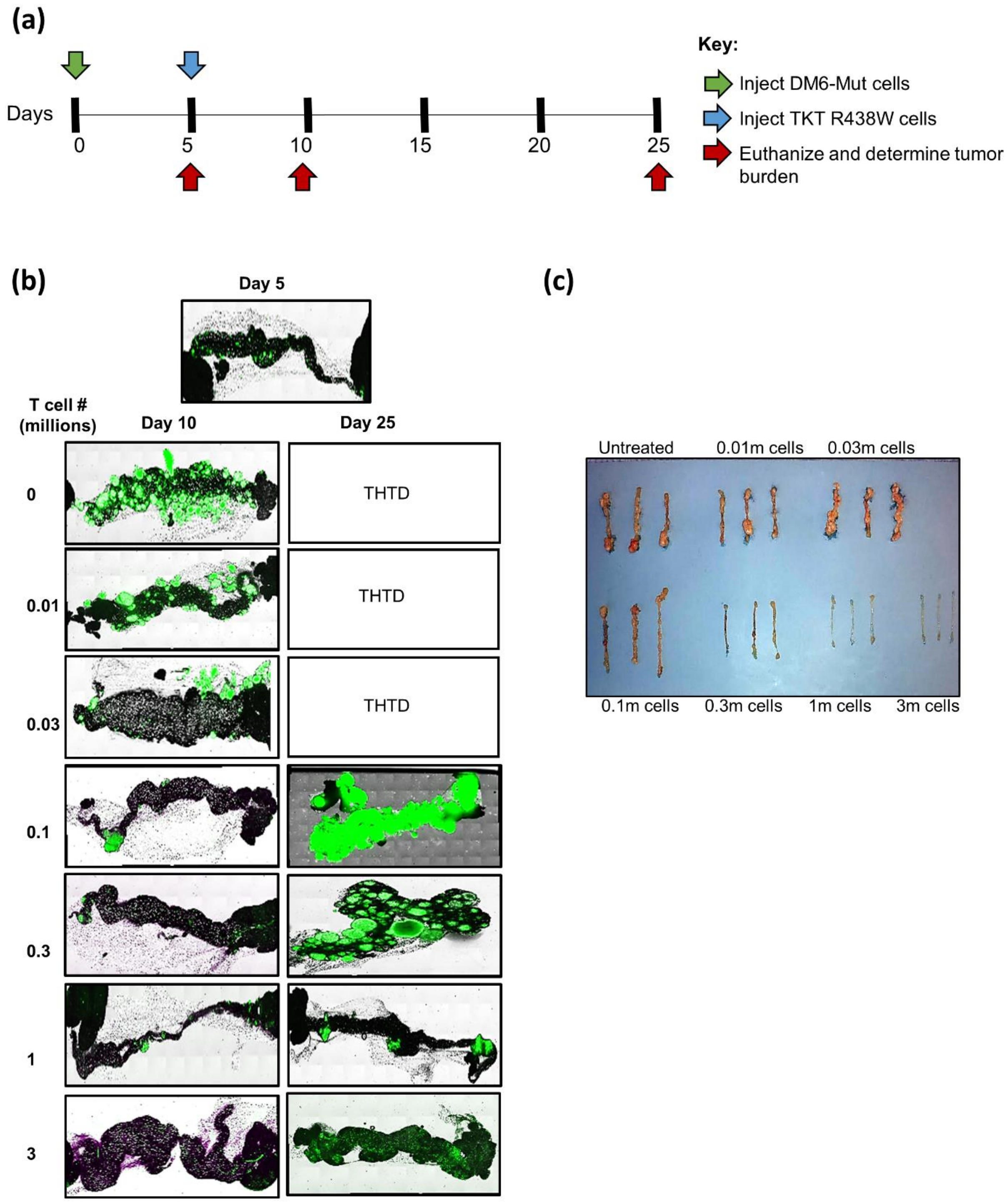

Supplementary Figure 4: Increasing the number of adoptively transferred T cells delays, but does not prevent tumor escape. **(a)** Experimental scheme indicating the timeline for injection of tumor cells (green arrow), TKT R438W cells (blue arrow) and estimation of tumor burden (red arrows). **(b)** Representative images of omental tumor burdens on the days 5, 10 and 25. **(c)** Gross images of omenta on day 25 from different groups are shown. n = 3.

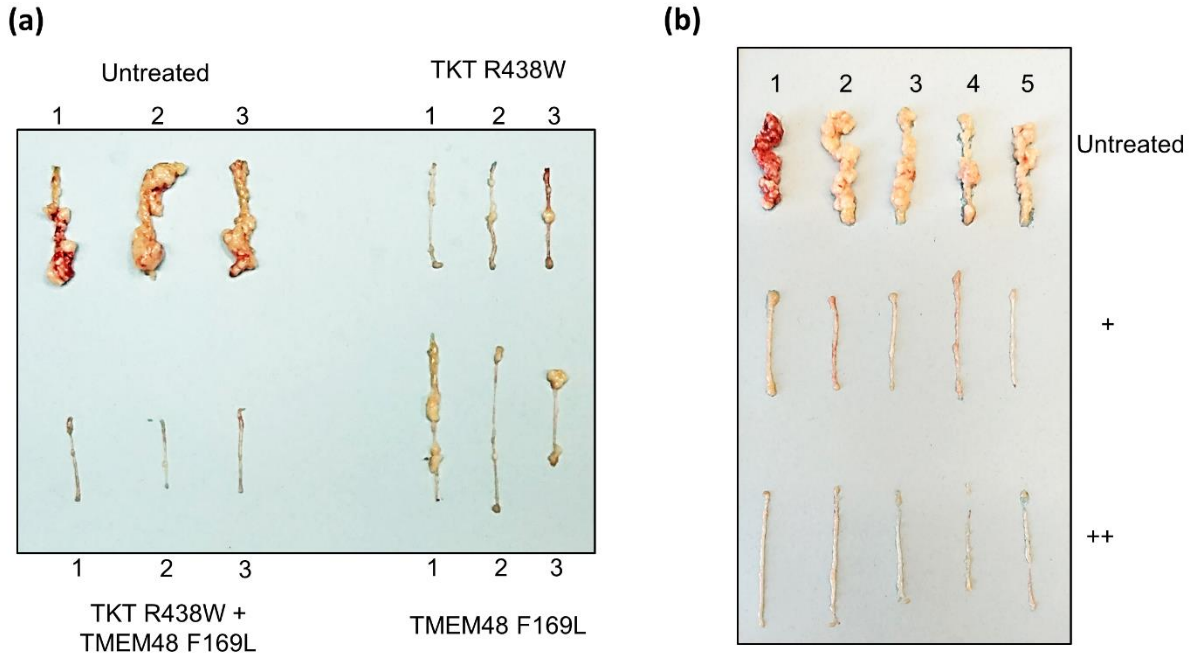

Supplementary Figure 5. Tumor burdens are reduced by combining tumor-specific T cells of different specificities or by repeated injections of T cells. Xenografts were established using DM6-Mut cells. **(a)** On day 5, TKT R438W cells and TMEM48 F169L cells were adoptively transferred individually or in combination in to the xenograft bearing mice. Gross images of omenta on day 25 from different groups are shown. **(b)** TKT R438W cells were injected on day 5 only (+), or on days 5 and 10 (++) into DM6-Mut xenografts. Gross images of omenta on day 25 from different groups are shown.

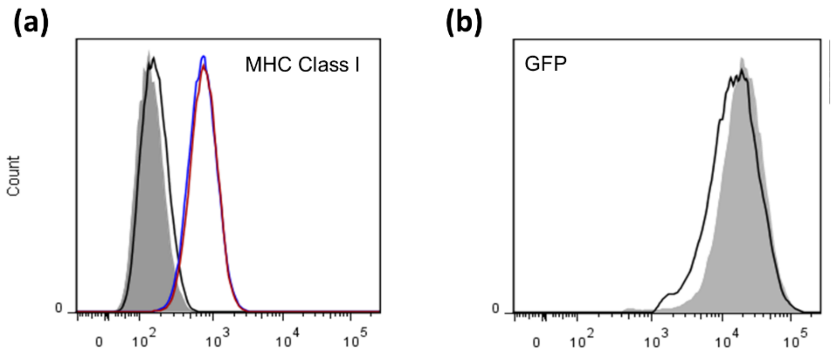

Supplementary Figure 6. Tumor escape is not due to loss of MHC or antigen **(a)** Levels of MHC class I on DM6-Mut cells from day 25 xenograft (red histogram) and cultured DM6-Mut cells (blue histogram). Unstained controls for day 25 DM6-Mut (black histogram) and cultured DM6-Mut cells (filled gray histogram) are shown. **(b)** GFP expression levels of DM6-Mut cells from day 25 xenograft (black histogram) and cultured DM6-Mut cells (filled gray histogram).

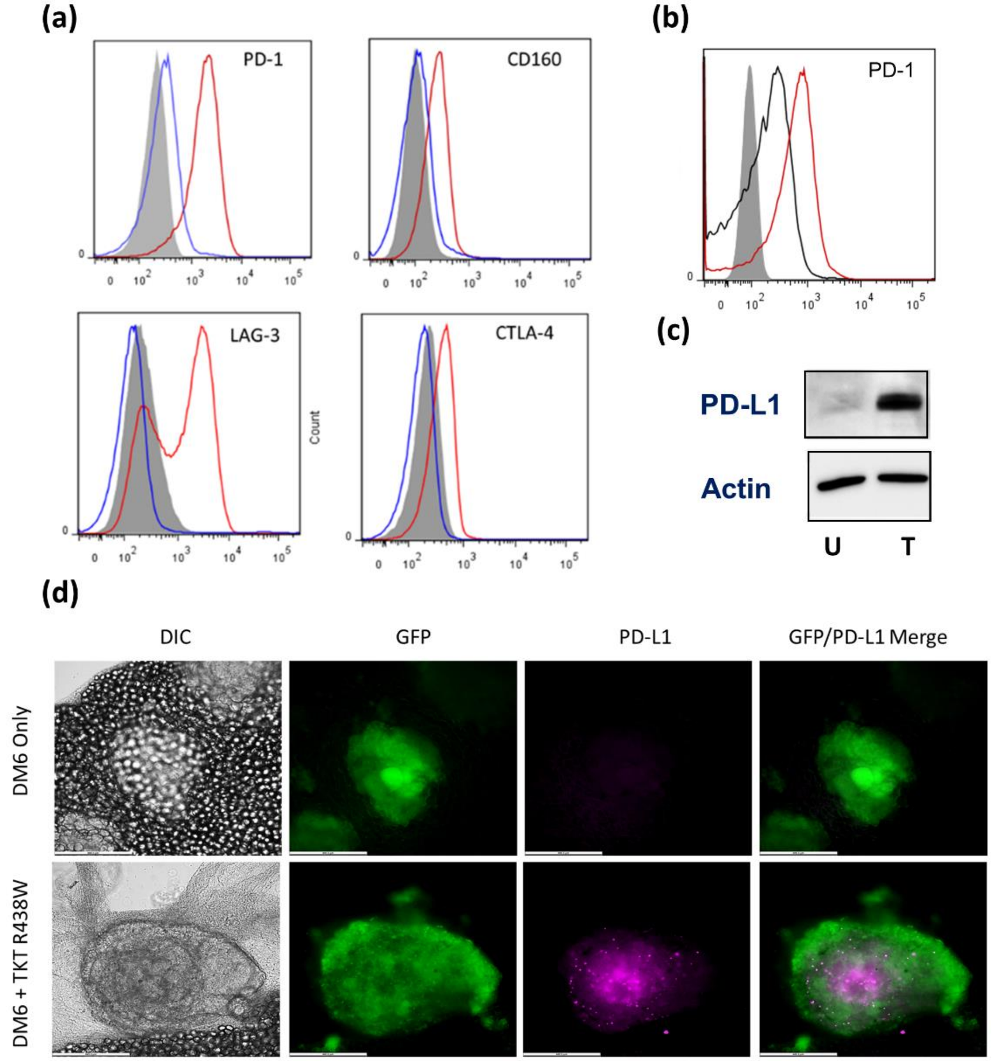

Supplementary Figure 7. Upregulation of checkpoint molecules is detected in TKT R438W and tumor target cells. **(a)** Expression of checkpoint molecules PD-1, CD160, LAG-3 and CTLA-4 when activated by T2 cells loaded with mutated (red histogram) or wild type peptide (blue histogram) for 3 days. Basal (day 0) levels of each marker are shown by the filled grey histograms. **(b)** PD-1 expression on TKT R438W cells ($CD3^+$ $CD8^+$ $Dextramer^+$; red histogram) isolated from DM6-Mut xenografts on day 10 compared to $CD3^+CD8^+$ T cells (black histogram) from peripheral blood. Unstained control is shown as filled gray histogram. **(c)** PD-L1 expression in DM6-Mut cells cultured for 48h without (U) or with (T) conditioned medium (from activated TKT R438W cells). **(d)** 2.5 x 10^6 DM6-Mut cells were injected i.p into NSG mice. These mice were either left untreated (DM6 only) or treated with 1 x 10^6 TKT R438W cells (DM6 + TKT R438W) on day 5. Omenta were harvested on day 25 and stained with Brilliant Violet 421-conjugated anti-PD-L1. Scale bar = 445.2 µm. Representative data shown (3 mice per group).

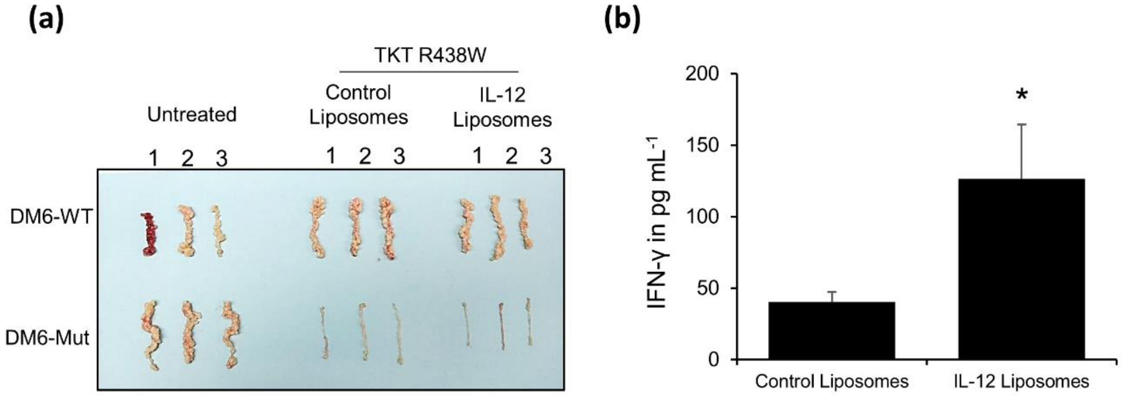

Supplementary Figure 8. Liposomally delivered IL-12 reduces tumor burden and correlates with higher circulating interferon. **(a)** 1 x 10^6 TKT R438W cells were injected on day 5 into xenografts established using DM6-WT or DM6-Mut cells. Control liposomes or liposomes with 20 µg IL-12 were injected on day 10. Gross images of omenta on day 25 from different groups are shown. **(b)** 1 x 10^6 TKT R438W cells were injected on day 5 into DM6-Mut xenografts, and control liposomes or liposomes with 20 µg IL-12 were injected on days 10, 15 and 20. Levels of IFN-γ in the plasma on day 15 were determined by ELISA. Data shown as Mean ± SEM. $n = 4$. $*P \leq 0.05$.

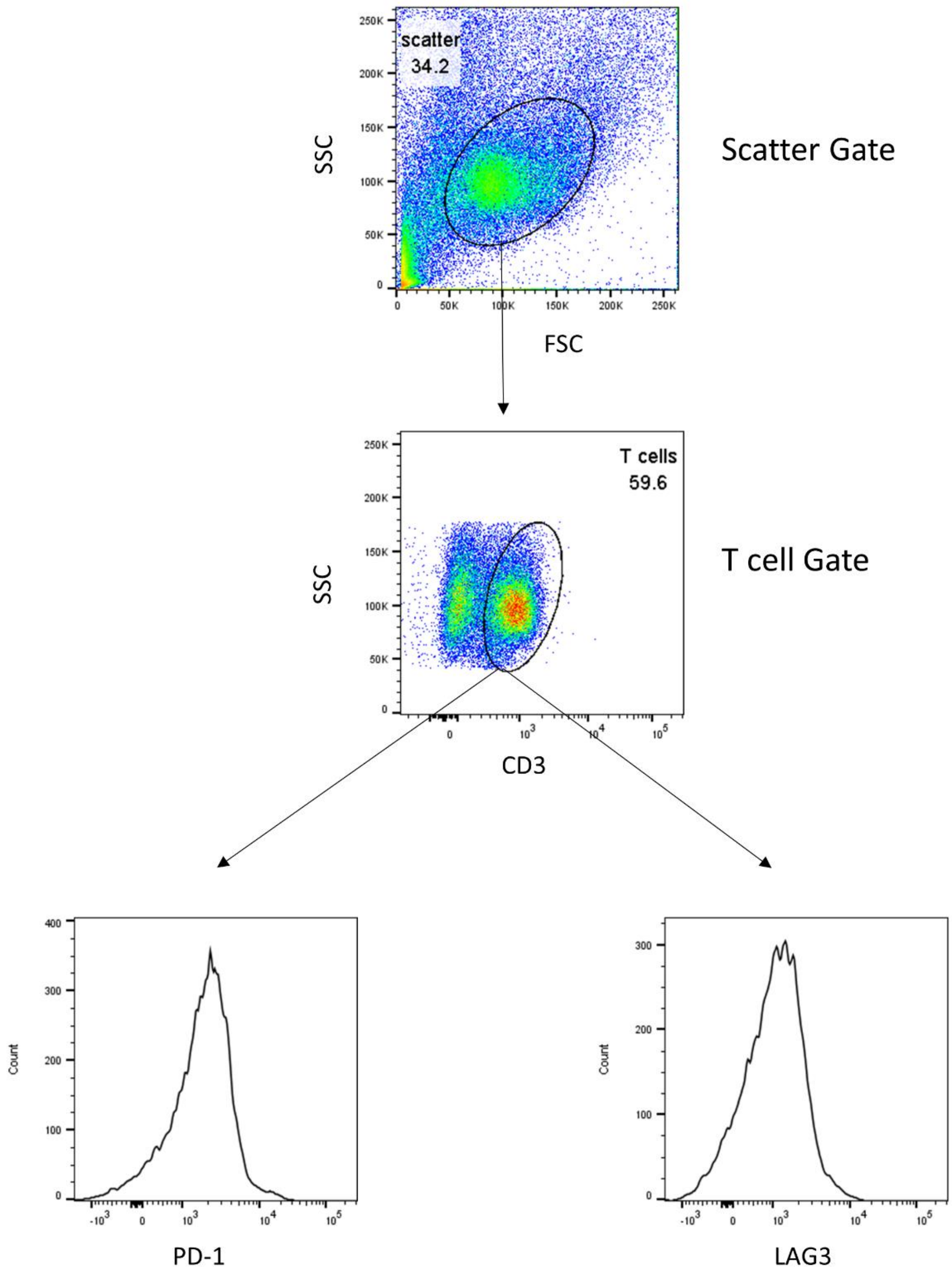

Supplementary Figure 9. Gating strategy for analysis of checkpoint molecules on T cells. A scatter gate was first applied to gate on lymphocytes, followed by gating on CD3+ cells (T cell gate). The expression of PD-1 and LAG3 was then determined on these gated CD3+ cells.

Supplementary Table 1. Day 25 Tumor burdens in DM6-Mut xenograft bearing mice treated with increasing numbers of T cells.

**Cell number (millions)**	**Day 5 Mean CTF#**	**Day 10 Mean CTF#**	**Fold change (D10/D5)**	**Day 25 Mean CTF#**	**Ascites Fluid (Day 25)**			**Metastatic Lesions (Day 25)**		
					**Mouse 1**	**Mouse 2**	**Mouse 3**	**Mouse 1**	**Mouse 2**	**Mouse 3**
0	6.74×10^{4}	4.3×10^{5}	6.37	THTD*	+	+	+	+	+	+
0.01	-	3.68×10^{5}	5.46	THTD*	+	+	+/-	+	+	+
0.03	-	1.37×10^{5}	2.03	THTD*	+	+/-	+	+	+	+
0.1	-	6.15×10^{4}	0.91	5.86×10^{9}	+/-	+/-	+/-	+	+	+
0.3	-	4.28×10^{4}	0.64	8.08×10^{5}	+	+/-	+	+	+	+
1	-	3.88×10^{4}	0.58	7.22×10^{4}	-	-	-	-	-	-
3	-	1.71×10^{4}	0.25	7.95×10^{4}	-	-	-	-	-	-

#Corrected Total Fluorescence
*Too high to determine accurately
(*n* = 3)

Supplementary Table 2. List of reagents used for flow cytometry.

Reagent	Fluorochrome	Manufacturer	Catalog #	Manufacturer Location
TKT R438W Peptide loaded HLA-A*02:01 Dextramers	PE	Immudex	WB3987-PE	Fairfax, VA
Anti-CD3	FITC	BD Biosciences	555332	San Jose, CA
	PE-Cy7		557851	
	AlexaFluor 647		557706	
	Pacific Blue		558117	
	PE		555333	
Anti-CD8	APC		555369	
	APC-Cy7		335805	
Anti-CTLA4	APC		555855	
Anti-HLA ABC	APC		555555	
Anti-PD1	PE		557946	
	APC	eBioscience	17-9969-42	San Diego, CA
Anti-CD69	PE-Cy7	BioLegend	877-246-5343	San Diego, CA
Anti-LAG-3	FITC	Enzo	ALX-804-806F-C100	Farmingdale, NY
Anti-CD160	FITC	AbD Serotec	MCA2238F	Oxford, UK
Sytox Red	-	Life Technologies	S34859	Grand Island, NY
Ghost Dye Red 710	-	Tonbo Biosciences	13-0871-T100	San Diego, CA
